# Factors associated with frequent buprenorphine / naloxone initiation in a national survey of Canadian emergency physicians

**DOI:** 10.1371/journal.pone.0297084

**Published:** 2024-02-05

**Authors:** Nathalie MacKinnon, Daniel Lane, Frank Scheuermeyer, Janusz Kaczorowski, Kathryn Dong, Aaron M. Orkin, Raoul Daoust, Jessica Moe, Gary Andolfatto, Michelle Klaiman, Justin Yan, Justin J. Koh, Kathryn Crowder, Paul Atkinson, David Savage, James Stempien, Floyd Besserer, Jason Wale, Andrew Kestler

**Affiliations:** 1 Department of Emergency Medicine, University of British Columbia, Vancouver, BC, Canada; 2 Department of Emergency Medicine, St. Paul’s Hospital, Vancouver, British Columbia, Canada; 3 Center for Health Evaluation and Outcome Sciences, Vancouver, British Columbia, Canada; 4 Université de Montréal, Montreal, Quebec, Canada; 5 Department of Emergency Medicine, University of Alberta, Edmonton, Alberta, Canada; 6 Department of Family & Community Medicine, University of Toronto, Toronto, Ontario, Canada; 7 Centre de Recherche de l’Hôpital Sacré-Coeur de Montréal, Montreal, Quebec, Canada; 8 British Columbia Centre for Disease Control, Vancouver, British Columbia, Canada; 9 Department of Medicine, University of Toronto, Toronto, Ontario, Canada; 10 Division of Emergency Medicine, Schulich School of Medicine & Dentistry, Western University, London, Ontario, Canada; 11 Department of Emergency Medicine, University of Saskatchewan, Saskatoon, Saskatchewan, Canada; 12 Department of Emergency Medicine, University of Calgary, Calgary, Alberta, Canada; 13 Department of Emergency Medicine, Dalhousie University, St. John, New Brunswick, Canada; 14 Division of Clinical Sciences, Northern Ontario School of Medicine, Thunder Bay, Ontario, Canada; 15 Department of Emergency Medicine, University of British Columbia, Prince George, British Columbia, Canada; 16 Department of Emergency Medicine, University of British Columbia, Victoria, British Columbia, Canada; 17 British Columbia Centre on Substance Use, Vancouver, British Columbia, Canada; Lahore College for Women University, PAKISTAN

## Abstract

**Objective:**

To identify individual and site-related factors associated with frequent emergency department (ED) buprenorphine/naloxone (BUP) initiation. BUP initiation, an effective opioid use disorder (OUD) intervention, varies widely across Canadian EDs.

**Methods:**

We surveyed emergency physicians in 6 Canadian provinces from 2018 to 2019 using bilingual paper and web-based questionnaires. Survey domains included BUP-related practice, demographics, attitudes toward BUP, and site characteristics. We defined frequent BUP initiation (the primary outcome) as at least once per month, high OUD prevalence as at least one OUD patient per shift, and high OUD resources as at least 3 out of the following 5 resources: BUP initiation pathways, BUP in ED, peer navigators, accessible addiction specialists, and accessible follow-up clinics. We excluded responses from sites with <50% participation (to minimize non-responder bias) and those missing the primary outcome. We used univariate analysis to identify associations between frequent BUP initiation and factors of interest, stratifying by OUD prevalence.

**Results:**

We excluded 3 responses for missing BUP initiation frequency and 9 for low response rate at one ED. Of the remaining 649 respondents from 34 EDs, 374 (58%) practiced in metropolitan areas, 384 (59%) reported high OUD prevalence, 312 (48%) had high OUD resources, and 161 (25%) initiated BUP frequently. Age, gender, board certification and years in practice were not associated with frequent BUP initiation. Site-specific factors were associated with frequent BUP initiation (high OUD resources [OR 6.91], high OUD prevalence [OR 4.45], and metropolitan location [OR 2.39],) as were individual attitudinal factors (willingness, confidence, and responsibility to initiate BUP.) Similar associations persisted in the high OUD prevalence subgroup.

**Conclusions:**

Individual attitudinal and site-specific factors were associated with frequent BUP initiation. Training to increase physician confidence and increasing OUD resources could increase BUP initiation and benefit ED patients with OUD.

## Introduction

Over 8000 Canadians died of apparent opioid toxicity in 2021 more than double the deaths compared to five years prior [1]. Initiation and maintenance of opioid agonist therapy (OAT) is an evidence-based strategy that reduces mortality for people with opioid use disorder (OUD) [2]. Globally, wide variation in OAT access and OAT dosing suggest there remains ample opportunity to improve OAT practices [3]. Given people with OUD have high attendance in emergency departments (EDs), EDs are an opportune setting to initiate first-line OAT, buprenorphine/naloxone (BUP) [4–6]. In the United States, ED-based BUP initiation doubled retention rates in addiction treatment compared to passive referral to addiction services alone [6] and these results have been replicated in Canada [7].

Because engaging patients in addictions treatment is challenging, ED providers do not capitalize on every opportunity to initiate BUP. Although two-thirds of emergency physicians are willing to initiate BUP, only one-quarter report doing so at least once a month [8]. While US-based research has identified relevant individual- and site-level predictors of BUP initiation in the ED [9, 10], Canadian data are lacking.

Using a national cross-sectional survey, we aimed to identify physician and site factors associated with frequent BUP initiation.

## Methods

### Study design, setting and time period

This cross-sectional survey is nested in a project of the Canadian Research Initiative in Substance Misuse (CRISM) to implement OAT in Canadian EDs. We conducted a nationwide paper and electronic survey of physicians at rural and urban EDs in 6 provinces from December 1, 2018 to November 30, 2019. We previously published full survey methodology [8], in accordance with the CHERRIES reporting checklist (https://www.equator-network.org). The questionnaire in English and French included items adapted from previously validated Likert scale questions on attitudes, as well as questions on individual and site-specific characteristics (questionnaire: S1 Appendix). Online and paper surveys contained an additional information and consent form that informed potential participants that completion was voluntary and that answering any question constituted implied consent. The study (and its implied consent mechanism) was approved by the University of British Columbia Ethics Review Board (H18-01744), and where applicable by local review boards in other provinces.

### Population

We included practicing emergency physicians who were fully-fledged members (not trainees, not locum tenens) of physician groups serving participating study sites. We excluded responses that were duplicates or were missing the primary outcome. To minimize non-responder bias, we decided a priori on a high-participation strategy for each site, including recruitment of physician champions and exclusion of responses from physician groups with under 50% response rate. The physician champion role entailed ensuring high group participation and having a capacity to respond to site-specific survey results.

### Data collection

Research staff collected paper surveys and entered responses into licensed Qualtrics (Provo, UT) CoreXM^TM^ survey software on a secure University of British Columbia (UBC) platform, while online participants entered responses directly into the software. Respondent IP addresses and demographics were reviewed to identify double entries.

### Outcome measures

The primary outcome was self-reported BUP initiation, defined as “frequent” if physicians prescribed or dispensed BUP for home-initiation or initiated BUP in the ED at least once per month. Exposure variables of interest included site-level variables (OUD prevalence, OUD-specific resources available, hospital academic affiliation, and large metropolitan area), and individual-level variables (demographics, emergency speciality certification, years in practice, and attitudes.) Academic affiliation was defined as a site hosting an emergency medicine training program, while a large metropolitan area was defined as having a population over 1 million. Attitude variables (responsibility, willingness, and confidence to initiate BUP) were modified from Likert to binary variables to facilitate interpretation (threshold ≥7/10, corresponding to responsible, willing, and confident.) The number of OUD resources available (peers, specialist consultation, BUP in ED, BUP initiation pathways, follow-up clinics) were added to create a score from 1 to 5. “High OUD resources” was defined as a score equal to or greater than the mean resource score. “High OUD prevalence” was defined as reporting seeing on average one or more patients per shift with OUD.

### Data analysis

We calculated odds ratios to measure associations between each exposure variable and frequent BUP initiation, using univariable logistic regression. A subgroup analysis was performed with physicians reporting high OUD prevalence to identify patterns among physicians with the highest potential for increasing BUP initiation. Analyses were conducted with Stata software, version 14.2.

### Sample size

We did not undertake a specific sample size calculation, instead prioritizing a high-participation (>50% physician group members) strategy per site, to gain a representative picture of physicians at each site and minimize non-responder bias. Because the survey was nested in a larger implementation project, we chose sites based on geographic distribution and presence of physician champions able and willing to implement changes based on survey results.

## Results

We received responses from physicians working in 35 EDs in 6 Canadian provinces, excluded 3 responses for missing primary outcome and 9 from one ED with low response rate, leaving a total of 649 respondents out of a potential 798 eligible participants (81%) working at 34 EDs. Overall, 245 (38%) were female, median practice duration was 6–10 years and median age category was 40–49. Of respondents, 384 (59%) reported high OUD prevalence in their practice, 374 (58%) practiced in metropolitan areas, and 481(74%) worked at an academic site. The median OUD resource score was 3, with 312 (48%) reporting high (greater than 3) OUD resources. Of physicians surveyed, 161(25%) reported initiating BUP frequently. There was no association between frequent BUP initiation and gender, age, certification, or years practicing (Table 1). Practice in large metropolitan area was associated with frequent BUP initiation, but academic affiliation was not. Physicians who reported more confidence and willingness to prescribe BUP, and those feeling responsibility to initiate BUP were more likely to initiate BUP. High OUD resources were associated with frequent initiation (OR 6.91, 95%CI: 4.26–11.21,) as was high OUD prevalence (OR 4.45, 95%CI: 2.80–7.06.).

**Table 1:** Factors associated with frequent buprenorphine/naloxone initiation among all physician respondents (N = 649).

Variables	Descriptive Data			Univariable Analysis	
	Overall, N = 649	Infrequent BUP initiation (%) N = 488*	Frequent BUP initiation (%) N = 161*	Odds Ratio (CI)	OR 95%CI
Female gender	245	188 (77%)	57 (23%)	0.87	0.60–1.27
Age category above median (≥50)	170	134 (79%)	36 (21%)	0.76	0.50–1.16
Years in practice above median (>10)	327	244 (75%)	83 (25%)	1.06	0.74–1.52
Certification					
CCFP or other FP (reference group)	50	41 (82%)	9 (18%)		
ABEM or other international EM certificate	15	10 (67%)	5 (33%)	2.28	0.61–8.53
CCFP-EM	342	258 (75%)	84 (25%)	1.48	0.69–3.19
FRCP	241	178 (74%)	63 (26%)	1.61	0.74–3.52
Willing to initiate BUP	441	306 (69%)	135 (31%)	3.26	2.00–5.32
Confident to initiate BUP	400	272 (68%)	138 (32%)	3.09	1.97–4.84
Responsibility to initiate BUP	401	284 (71%)	117 (29%)	2.07	1.36–3.14
Academic Affiliation	481	354(73%)	127(26%)	1.41	0.92–2.17
Large metropolitan area (>1 million pop.)	374	257(69%)	117 (31%)	2.39	1.61–3.55
High OUD resources (greater than median, >3)	312	185 (59%)	127 (41%)	6.91	4.26–11.21
High OUD prevalence (≥1 OUD patient/shift)	384	251 (65%)	133 (35%)	4.45	2.80–7.06

Abbreviations: OUD: opioid use disorder BUP: buprenorphine/naloxone, CCFP: Canadian College of Family Practice, CCFP-EM: CCFP with additional competency in Emergency Medicine, ABEM: American Board of Emergency Medicine, FRCP: Fellow of the Royal College of Physicians

*Frequent BUP initiation: prescribing, dispensing, or administering buprenorphine/naloxone for initiation at least once per month

In the subgroup analysis of 384 physicians who reported high OUD prevalence in their practice, 133 (35%) initiated BUP frequently. OUD specific resources, confidence, willingness, sense of responsibility and practice in a large metropolitan area remained associated with frequent BUP initiation (Table 2).

**Table 2:** Factors associated with frequent buprenorphine/naloxone initiation among physicians reporting high prevalence opioid use disorder in their practice (N = 384).

Variables	Descriptive Data			Univariable Analysis	
	Overall, N = 384	Infrequent BUP initiation (%) N = 251	Frequent BUP initiation (%) N = 133	Odds Ratio	OR 95%CI
Female gender	139	91(65%)	48(35%)	0.99	0.64–1.54
Age category above median (≥50)	105	72(69%)	33(31%)	0.76	0.50–1.16
Years in practice above median (>10)	193	122(63%)	71(37%)	1.21	0.79–1.85
Certification					
CCFP or other FP (reference group)	20	13 (65%)	7 (35%)		
ABEM or other international EM certificate	8	4 (50%)	4 (50%)	1.86	0.34–10.27
CCFP-EM	187	122 (65%)	65 (35%)	0.99	0.38–2.61
FRCP	168	111 (66%)	57 (34%)	0.95	0.36–2.53
Willing to initiate BUP	279	168(60%)	111(40%)	2.54	1.46–4.44
Confident to initiate BUP	258	151 (59%)	107 (41%)	2.63	1.55–4.44
Responsibility to initiate BUP	242	146 (60%)	96(40%)	2.16	1.33–3.51
Academic Affiliation	308	192(62%)	116(38%)	2.10	1.16–3.79
Large metropolitan area (>1 million pop.)	217	119 (55%)	98 (45%)	3.11	1.93–4.99
High OUD resources (greater than median, >3)	214	109 (51%)	105 (49%)	5.53	3.18–9.62

Abbreviations: OUD: opioid use disorder BUP: buprenorphine/naloxone, CCFP: Canadian College of Family Practice, CCFP-EM: CCFP with additional competency in Emergency Medicine, ABEM: American Board of Emergency Medicine, FRCP: Fellow of the Royal College of Physicians

*Frequent BUP initiation: prescribing, dispensing, or administering buprenorphine/naloxone for initiation at least once per month

## Discussion

### Main findings

In this pan-Canadian survey of emergency physicians, we found that a frequent BUP initiation is strongly associated with the site-level factors of OUD prevalence and OUD-related resources available in the ED. Frequent BUP initiation was also associated with individual physician willingness, perceived responsibility, and confidence in prescribing BUP, but not with individual physician demographics. In the subgroup of physicians who reported high OUD prevalence in their practice, similar patterns related to resources and attitudes persisted.

### Comparison with existing literature

While an American study suggested that younger emergency doctors are more likely to approve ED BUP initiation [9], we did not find the same association in Canada. Our results show an association between high prescribing rates and ED resources, corroborating commonly cited barriers of time, resources, and knowledge in the ED [9, 10]. In the United States, attitudes may be a stronger barrier than lack of follow-up clinics and training [11]. We found physician responsibility to initiate BUP to be associated with high prescribing. However, most respondents in our sample felt responsible for initiating BUP, contrasting a commonly cited barrier from the United States that BUP initiation is not within the scope of ED practice [9, 10]. We further found physician confidence in initiating BUP affects its use in practice, as previously noted elsewhere [10, 11]. Fortunately, lack of confidence may be amenable to intervention. One ED study demonstrated fluctuating efficacy with subsequent improvement with “just-in time” re-training: reminding physicians how to prescribe BUP within their context caused an increase in prescribing overall [12].

### Strengths/limitations

Our survey achieved more than 50% response rate in all participating EDs except one (which was excluded from the analysis), higher than most ED physician surveys and high enough to minimize non-responder bias risk. We also recognize the limitations of our study sample: site selection that may have under-represented some provinces or some types of physician groups, respondent self-selection, and risk of recall and social desirability bias, all which could have impacted results, although the direction and magnitude of bias are unclear. Our cut-off points for converting Likert scales to dichotomous variables were based on reasonable assumptions but were to some degree arbitrary and result in some loss of data richness. The small size of some sites made cluster analysis impractical and thus difficult to know whether all factors had similar associations with the primary outcome across sites. Many factors examined are correlated and therefore make inferences on causation more difficult. Our results can only establish hypotheses.

### Clinical implications

Many factors associated with low buprenorphine initiation frequency are amenable to intervention. EDs can increase available addiction-related resources to increase BUP use in clinical practice. Many of these resources, such as widespread availability of BUP, standard BUP initiation pathways, access to follow-up (in-person or virtual) can benefit from a regional approach that decreases burden on individual EDs, as exemplified in Alberta [7]. This regional approach may be particularly helpful in smaller metropolitan regions and rural sites, noted to have lower BUP initiation rates even when controlling for OUD prevalence. Individual EDs and regional authorities can additionally implement targeted education to boost provider confidence with BUP.

### Research implications

Despite the publication of national ED position statements and recommended OUD training [4], more research could elucidate the causative pathways leading from individual and institutional factors to actual BUP initiation and could investigate the implementation tools needed to translate an established responsibility to initiate BUP into patient care at the ED bedside.

## Conclusion

OUD prevalence, physician responsibility, physician confidence, and ED OUD-specific resources are related to frequent buprenorphine initiation. Increasing ED-specific OAT training and OUD-related resources may help EDs increase BUP initiation to better serve patients at high mortality risk.

## Supporting information

S1 Appendix Study questionnaire. (DOCX)

S1 Dataset Study minimal dataset. (XLS)
