# Supplementary material for: Factors associated with frequent buprenorphine / naloxone initiation in a national survey of Canadian emergency physicians
Source: PLoS One. 2024 Feb 5;19(2):e0297084. doi: 10.1371/journal.pone.0297084 (PMC10843078; doi:10.1371/journal.pone.0297084)
Supplement: S1 Appendix — (DOCX) [file pone.0297084.s001.docx]

**Emergency physician attitudes and practices on prescribing buprenorphine / naloxone**

The opioid crisis is one of the most significant public health problems of this generation. While there are a great many factors that contribute to this public health emergency, there are a number of steps physicians working in emergency departments can take to assist patients with opioid use disorder.

Buprenorphine/naloxone (Suboxone) can be initiated in the emergency department and improves addiction follow-up care. It decreases overdose and all-cause mortality. This survey seeks to better understand attitudes and prescribing practices related to emergency department initiation.

Because questions below have been adapted from different validated surveys, they contain scales with differing numeric values. In the questionnaire below, BNX refers to buprenorphine/naloxone.

**Section 1. Demographic Information**

Item 1. Which gender do you identify with?

Male  Female  Other:_________________

Item 2. What is your age category?

less than 30 years

30-39 years

40-49 years

50+ years

Item 3. What is your certification? *(Select all that apply)*

FRCP

ABEM

CCFP-EM

CCFP

Other FP: _________

Other: ____________

Item 4. How many years have you been practicing emergency medicine? *(Since completing training)*

0-2 years

3-5 years

5-10 years

greater than 10 years

**Section 2. Physician Practice Characteristics**

Item 1. On average, in your ED practice, how often do you do the following?

(Please circle the best choice for each item)

| 1a. Treat patients who use non-medical opioids | Never | Once or more in your career | Once or more per year | Once or more per month | Once or more per shift |
| --- | --- | --- | --- | --- | --- |
| 1b. Order BNX (directly or via Addictions consult) for ED initiation | Never | Once or more in your career | Once or more per year | Once or more per month | Once or more per shift |
| 1c. Prescribe or dispense BNX for a home start (community initiation) | Never | Once or more in your career | Once or more per year | Once or more per month | Once or more per shift |

Item 2. What BNX-related resources are available for physicians in your ED?

(Please circle your choice for each item, yes/no):

| 2a. Timely access to an addiction’s specialist either in person or via telephone | Yes | No |
| --- | --- | --- |
| 2b. Access to hospital/regional pathway for BNX initiation in the ED | Yes | No |
| 2c. BNX available for order in your ED/hospital | Yes | No |
| 2d. BNX to-go packs for home initiation | Yes | No |
| 2e. Peer support workers who can meet a patient with opioid use in your ED | Yes | No |
| 2f. Access to clinics or family physicians willing to do ED BNX continuation | Yes | No |

Item 3: Given your current resources and presented with the following scenario tomorrow in your ED, how likely would you be to: (Please circle your choice for each item, likely/not likely):

| 3a. Order BNX for a patient in acute opioid withdrawal | Likely | Not likely |
| --- | --- | --- |
| 3b. Prescribe BNX for a home start for a patient not in acute opioid withdrawal | Likely | Not likely |
|  |  |  |

**Section 3. Attitudes on Opioid Harm Reduction**

Item 1. How WILLING are you to do the following for your patients?

(Please circle your choice, on a scale of 1-10*)*:

|  | Not Willing | | | Neutral | | | | Very willing | | | |
| --- | --- | --- | --- | --- | --- | --- | --- | --- | --- | --- | --- |
| 1a. Provide naloxone (Narcan) kits to people who use opioids | 1 | 2 | 3 | 4 | 5 | 6 | 7 | | 8 | 9 | 10 |
| 1b. Conduct brief screening & education about unhealthy substance use, including alcohol | 1 | 2 | 3 | 4 | 5 | 6 | 7 | | 8 | 9 | 10 |
| 1c. Refer to a detox program or an addiction clinic | 1 | 2 | 3 | 4 | 5 | 6 | 7 | | 8 | 9 | 10 |
| 1d. Refer to a needle exchange/syringe access program | 1 | 2 | 3 | 4 | 5 | 6 | 7 | | 8 | 9 | 10 |
| 1e. Start BNX in the ED | 1 | 2 | 3 | 4 | 5 | 6 | 7 | | 8 | 9 | 10 |
| 1f. Prescribe/dispense BNX for home starts | 1 | 2 | 3 | 4 | 5 | 6 | 7 | | 8 | 9 | 10 |

Item 2. Provided your ED had the necessary resources, how CONFIDENT are you in your ABILITY to do the following for your patients? (Please circle your choice, on a scale of 1-10):

|  | Not at all Confident | | | Neutral | | | | Very confident | | |
| --- | --- | --- | --- | --- | --- | --- | --- | --- | --- | --- |
| 2a. Provide naloxone (Narcan) kits to people who use opioids | 1 | 2 | 3 | 4 | 5 | 6 | 7 | 8 | 9 | 10 |
| 2b. Conduct brief screening & education about unhealthy substance use, including alcohol | 1 | 2 | 3 | 4 | 5 | 6 | 7 | 8 | 9 | 10 |
| 2c. Refer to a detox program or an addiction clinic | 1 | 2 | 3 | 4 | 5 | 6 | 7 | 8 | 9 | 10 |
| 2d. Referral to needle exchange/syringe access program | 1 | 2 | 3 | 4 | 5 | 6 | 7 | 8 | 9 | 10 |
| 2e. Start BNX in the ED | 1 | 2 | 3 | 4 | 5 | 6 | 7 | 8 | 9 | 10 |
| 2f. Prescribe/dispense BNX for home starts | 1 | 2 | 3 | 4 | 5 | 6 | 7 | 8 | 9 | 10 |

Item 3. Provided your ED has/had BNX, how CONFIDENT are you in your ABILITY to perform specific aspects of BNX initiation? (Please circle your choice, on a scale from 1-10):

|  | Not at all Confident | | | Neutral | | | | Very confident | | | |
| --- | --- | --- | --- | --- | --- | --- | --- | --- | --- | --- | --- |
| 3a. Screen patients to determine whether or not they would benefit from BNX | 1 | 2 | 3 | 4 | 5 | 6 | 7 | | 8 | 9 | 10 |
| 3b. Initiate a discussion with at-risk patients regarding BNX initiation | 1 | 2 | 3 | 4 | 5 | 6 | 7 | | 8 | 9 | 10 |
| 3c. Assess severity of withdrawal to determine if candidate for initiation of BNX in the ED | 1 | 2 | 3 | 4 | 5 | 6 | 7 | | 8 | 9 | 10 |
| 3d. Administer BNX to a patient in opioid withdrawal &provide prescription for continuation | 1 | 2 | 3 | 4 | 5 | 6 | 7 | | 8 | 9 | 10 |
| 3e. Discharge a patient with a prescription or to-go pack for a BNX home start | 1 | 2 | 3 | 4 | 5 | 6 | 7 | | 8 | 9 | 10 |
| 3f. Arrange for a follow-up visit after ED BNX initiation | 1 | 2 | 3 | 4 | 5 | 6 | 7 | | 8 | 9 | 10 |

Item 4. How SIGNIFICANT are the following barriers to your initiating BNX in the ED? (Please circle your choice, on a scale of 1-5).

|  | Not significant | | Moderately significant | | | Extremely significant | |
| --- | --- | --- | --- | --- | --- | --- | --- |
| 4a. Lack of time during the clinical encounter | 1 | 2 | | 3 | 4 | | 5 |
| 4b. Lack of adequate training to initiate BNX | 1 | 2 | | 3 | 4 | | 5 |
| 4c. Limited knowledge of research to support ED initiation of BNX | 1 | 2 | | 3 | 4 | | 5 |
| 4d. Lack of hospital or ED administrative support for BNX | 1 | 2 | | 3 | 4 | | 5 |
| 4e. Lack of ED rooms to initiate BNX | 1 | 2 | | 3 | 4 | | 5 |
| 4f. Lack of adequate outpatient follow-up options | 1 | 2 | | 3 | 4 | | 5 |
| 4g. Other:___________________________________ | 1 | 2 | | 3 | 4 | | 5 |

Item 5. How would the following IMPACT the likelihood of your starting patients on BNX? (Please circle your choice, on a scale of 1-10):

|  | No  Impact | | | Moderate  Impact | | | | Strong  Impact | | |
| --- | --- | --- | --- | --- | --- | --- | --- | --- | --- | --- |
| 5a. Strong evidence that prescribing BNX decreases overdose mortality | 1 | 2 | 3 | 4 | 5 | 6 | 7 | 8 | 9 | 10 |
| 5b. If professional organizations’ guidelines recommended prescribing BNX in the ED | 1 | 2 | 3 | 4 | 5 | 6 | 7 | 8 | 9 | 10 |
| 5c. If ED leaders where you work recommended prescribing BNX | 1 | 2 | 3 | 4 | 5 | 6 | 7 | 8 | 9 | 10 |
| 5d. If it were common practice in the ED where you work | 1 | 2 | 3 | 4 | 5 | 6 | 7 | 8 | 9 | 10 |
| 5e. If ED nurses where you work supported ED BNX and assisted with initiation | 1 | 2 | 3 | 4 | 5 | 6 | 7 | 8 | 9 | 10 |
| 5f. If the ED had specialized staff to assist with BNX initiation (pharmacists, addiction nurses, social workers, peer educators etc.) | 1 | 2 | 3 | 4 | 5 | 6 | 7 | 8 | 9 | 10 |
| 5g. Timely in-person or telephone access to an addictions specialist | 1 | 2 | 3 | 4 | 5 | 6 | 7 | 8 | 9 | 10 |
| 5h. Local clinical pathways covering initial assessment, BNX initiation, & follow-up | 1 | 2 | 3 | 4 | 5 | 6 | 7 | 8 | 9 | 10 |
|  |  |  |  |  |  |  |  |  |  |  |

Item 6: In your opinion, what impact do you think ED initiation of BNX (in ED or via home start) will have on the following: (Please circle your choice, on a scale of 1-5).

|  | Large increase | | No  change | | | Large decrease | |
| --- | --- | --- | --- | --- | --- | --- | --- |
| 6a. Deaths due to overdose | 1 | 2 | | 3 | 4 | | 5 |
| 6b. Opioid use overall | 1 | 2 | | 3 | 4 | | 5 |
| 6c. Frequency of 911 calls for opioid overdose | 1 | 2 | | 3 | 4 | | 5 |
| 6d. ED visits for opioid overdose | 1 | 2 | | 3 | 4 | | 5 |
|  |  |  | |  |  | |  |

**Section 4. General Attitudes on Addictions and Harm Reduction**

Item 1. What level of RESPONSIBILITY do EDs and emergency physicians have to perform the following harm reduction or public health interventions? (Please circle your choice, on a scale of 1-10)

|  | No responsibility | | | Some  responsibility | | | | | Major responsibility | | | |
| --- | --- | --- | --- | --- | --- | --- | --- | --- | --- | --- | --- | --- |
| 1a. HIV Screening | 1 | 2 | 3 | | 4 | 5 | 6 | 7 | 8 | | 9 | 10 |
| 1b. Screening & counseling for interpersonal violence | 1 | 2 | 3 | | 4 | 5 | 6 | 7 | 8 | | 9 | 10 |
| 1c. Screening & education for seatbelt use | 1 | 2 | 3 | | 4 | 5 | 6 | 7 | 8 | | 9 | 10 |
| 1d. Naloxone kits to treat opioid overdoses | 1 | 2 | 3 | | 4 | 5 | 6 | 7 | 8 | | 9 | 10 |
| 1e. Brief screening & education about unhealthy substance use, including alcohol | 1 | 2 | 3 | | 4 | 5 | 6 | 7 | 8 | | 9 | 10 |
| 1f. Prescriptions for emergency contraception (Plan B) | 1 | 2 | 3 | | 4 | 5 | 6 | 7 | 8 | | 9 | 10 |
| 1g. Referral to detox programs & addiction clinics | 1 | 2 | 3 | | 4 | 5 | 6 | 7 | 8 | | 9 | 10 |
| 1h. Smoking cessation counseling | 1 | 2 | 3 | | 4 | 5 | 6 | 7 | | 8 | 9 | 10 |
| 1i. Referral to needle exchange/syringe access program | 1 | 2 | 3 | | 4 | 5 | 6 | 7 | | 8 | 9 | 10 |
| 1j. BNX initiation for opioid use disorder | 1 | 2 | 3 | | 4 | 5 | 6 | 7 | | 8 | 9 | 10 |

Item 2. Please indicate how much you agree or disagree with the following statements as they relate to addictions (Please circle your choice, on a scale of 1-5).

|  | Strongly Disagree | | Neutral | | | Strongly Agree | |
| --- | --- | --- | --- | --- | --- | --- | --- |
| 2a. Addiction is a chronic medical illness similar to asthma, diabetes, and hypertension | 1 | 2 | | 3 | 4 | | 5 |
| 2b. Addiction is the result of changes in brain neuro-circuitry | 1 | 2 | | 3 | 4 | | 5 |
| 2c. Addiction is influenced by psychological and environmental factors | 1 | 2 | | 3 | 4 | | 5 |
|  |  |  | |  |  | |  |

Item 3. There are a range of feelings and thoughts about working with patients with substance use. Please indicate how much you agree or disagree with the following statements (Please circle your choice, on a scale from 1-7).

|  | Strongly Disagree | | Neutral | | | Strongly Agree | |  |
| --- | --- | --- | --- | --- | --- | --- | --- | --- |
| 3a. “I feel that there is little I can do to help people who use drugs.” | 1 | 2 | 3 | 4 | 5 | 6 | 7 | |
| 3b. “I feel that I am able to work with people who use drugs as well as other client groups.” | 1 | 2 | 3 | 4 | 5 | 6 | 7 | |
| 3c. “I am inclined to feel that I am a failure with people who use drugs.” | 1 | 2 | 3 | 4 | 5 | 6 | 7 | |
| 3d. “I have less respect for people who use drugs than for most other patients I work with.” | 1 | 2 | 3 | 4 | 5 | 6 | 7 | |
| 3e. “I often feel uncomfortable when working with people who use drugs.” | 1 | 2 | 3 | 4 | 5 | 6 | 7 | |
| 3f. “One can get satisfaction from working with people who use drugs.” | 1 | 2 | 3 | 4 | 5 | 6 | 7 | |
| 3g. “It is rewarding to work with people who use drugs.” | 1 | 2 | 3 | 4 | 5 | 6 | 7 | |
| 3h. “I feel I can understand people who use drugs.” | 1 | 2 | 3 | 4 | 5 | 6 | 7 | |
|  |  |  |  |  |  |  |  | |

THANK YOU FOR YOUR PARTICIPATION!
